# What does ‘quality’ add? Towards an ethics of healthcare improvement

**DOI:** 10.1136/medethics-2019-105635

**Published:** 2019-11-15

**Authors:** Alan Cribb, Vikki Entwistle, Polly Mitchell

**Affiliations:** 1 Centre for Public Policy Research, King’s College London, London, UK; 2 Centre for Biomedical Ethics, National University of Singapore, Singapore

**Keywords:** healthcare improvement, quality, ethics, ethics quality

## Abstract

In this paper, we argue that there are important ethical questions about healthcare improvement which are underexplored. We start by drawing on two existing literatures: first, the prevailing, primarily governance-oriented, application of ethics to healthcare ‘quality improvement’ (QI), and second, the application of QI to healthcare ethics. We show that these are insufficient for ethical analysis of healthcare improvement. In pursuit of a broader agenda for an ethics of healthcare improvement, we note that QI and ethics can, in some respects, be treated as closely related concerns and not simply as externally related agendas. To support our argument, we explore the gap between ‘quality’ and ‘ethics’ discourses and ask about the possible differences between ‘good quality healthcare’ and ‘good healthcare’. We suggest that the word ‘quality’ both adds to and subtracts from the idea of ‘good healthcare’, and in particular that the technicist inflection of quality discourses needs to be set in the context of broader conceptualisations of healthcare improvement. We introduce the distinction between quality as a measurable property and quality as an evaluative judgement, suggesting that a core, but neglected, question for an ethics of healthcare improvement is striking the balance between these two conceptions of quality.

Healthcare quality is a major policy and public concern and healthcare improvement is a high-profile feature of professional practice and debate. However, these themes are relatively neglected in the healthcare ethics literature. This is surprising given the substantive importance of the area in two respects. First, is the question of scale—very many people are involved in the quality ‘business’ which, like public health, impacts on the lives of populations and communities as well as professional-patient dyads. Second, is the question of focus—this is an area of health policy and practice that is expressly concerned with both making determinations about, and pursuing or underpinning, ‘good’ healthcare, something which can hardly be seen as marginal to healthcare ethics.

In this paper, we argue that there are important ethical questions about healthcare improvement which are underexplored. We start by drawing on two existing literatures: first, the prevailing, primarily governance-oriented, application of ethics to healthcare ‘quality improvement’ (QI), and second, the application of QI to healthcare ethics. We show that these are insufficient for ethical analysis of healthcare improvement. In pursuit of a broader agenda for an ethics of healthcare improvement, we note that QI and ethics can, in some respects, be treated as closely related concerns and not simply as externally related agendas. To support our argument, we explore the gap between ‘quality’ and ‘ethics’ discourses and ask about the possible differences between ‘good quality healthcare’ and ‘good healthcare’. We suggest that the word ‘quality’ both adds to and subtracts from the idea of ‘good healthcare’, and in particular that the technicist inflection of quality discourses needs to be set in the context of broader conceptualisations of healthcare improvement.

## Applying ethics to QI

Healthcare improvement is a well-established domain, although one with fuzzy boundaries. QI, in particular, is a professional or quasi-professional field: some health services staff have designated QI roles, and there are distinct QI projects and practices with an extensive supporting body of research and education. The broader domain of healthcare improvement might refer to the work of many others, not least the regulatory, guidance, inspection and educational agencies that contribute to the quality of health systems and healthcare practices, and sometimes do so explicitly under the description of QI.

The existing ‘QI ethics’ literature mainly revolves around the ethical oversight of designated QI projects.^1–3^ It has been generated by the perceived need to classify QI projects in relation to healthcare research projects, given that the latter are subject to careful ethical governance and demanding data protection legislation. Sometimes the relative distinctiveness of QI is emphasised. For example, that QI is different to research because it is a routine part of healthcare practice, and thus an inherent obligation, rather than an optional separate activity for both health professionals and patients. Or that QI is different to research because it is not centred on the production of generalised knowledge but rather on immediate local benefit. To make these distinctions, Lynn *et al* 4 rely on a definition of QI activities as ‘systematic, data-guided activities designed to bring about immediate improvements in healthcare delivery in particular settings’ (p. 667). Other conceptions of QI that argue that it can work towards and implement generalisable lessons tend not to make sharp distinctions between research and QI but treat QI as characteristically including both practice and research components.^5^

This work highlights that, whether or not QI should be routinely subject to exactly the same ethics governance as research, some kind of ethical oversight and accountability is needed for QI activity. QI creates the same kinds of ethical challenges as any other kind of healthcare-related activity; for example, it can produce harm, waste resources, treat individuals and groups unfairly, fail to respect privacy and so on. Some authors and agencies have thus proposed principles and safeguards to protect human subjects from QI activity, and proportionate forms of accountability to underpin these.^4^ As part of a set of concerns that include attention to such things as privacy and appropriate consent processes, they argue that ‘(t)he gains from a QI activity should justify the resources spent and the risks imposed on participants’ and ‘(a) QI activity should be designed to limit risks while maximising potential benefits and to ensure that risks to an individual human participant are balanced by expected benefits to the participant and to society’ (p. 668).

Although it involves some simplification, we suggest that QI is currently often framed as a set of techniques, or a ‘technology’, and the business of ethically appraising QI tends to be treated as a form of technology appraisal with utilitarian calculation at its backbone. In the above quotations, for example, QI activities are presented as being directed towards ‘gains’ or ‘benefits’ which have to outweigh the associated ‘resources’ and ‘risks’. This is, of course, accompanied by attention to important side-constraints such as consent. But in a guide to managing QI ethics,^3^ the need for ethical oversight is itself explained by the fact that QI activities risk creating physical or psychological harms from which people need protection rather than by reference to any questioning of the values inherent in QI purposes or processes. In practice, of course, QI activities can be ethically analysed like any other kind of healthcare activity. They rest on assumptions about what matters and they deploy specific methods. For example, even a ‘routine’ intervention that consists of guidance designed to underpin the clinical effectiveness of a treatment raises ethical questions about the ways in which effectiveness is defined and the broader ethical effects and defensibility of clinical guidance. In other words, the literature on QI ethics governance does comparatively little to question the broader ethical constitution of QI; for example, to acknowledge or unpack the full range of goods that are served by, or embodied in, QI activities, or to reflect on the ethical status of the practice of QI.

## Applying QI to ethics

There are, of course, many possible conceptions of, and dimensions of, quality.^6–9^ In healthcare contexts, quality is often equated with some relatively focused concerns—most frequently that healthcare practices should be effective and safe—but it can be extended to include an indeterminate number of other considerations. For example, the highly influential Institute of Medicine account of quality includes six dimensions—effectiveness, safety, timeliness, efficiency, equity and patient-centredness.^8^ One small part of the vast healthcare quality literature focuses on the idea of ‘ethics quality’, that is, on ethics as a dimension of quality.

The idea of ethics quality has been adopted by the US Veterans Health Administration (VHA) in order to ensure that ethics—specifically, attention to the underpinning and enactment of appropriate ethical standards—is included in the quality assurance and improvement efforts of their healthcare institutions. An important founding text in this field is Susan M Wolf’s 1994 paper ‘Quality Assessment of Ethics in Health Care’.^10^ Wolf argues that the rise of bioethics in the USA in the 1970s and 1980s coincided with the rise of healthcare quality assurance. The latter was needed, among other reasons she cites, to protect standards and serve public accountability in an era of cost-containment. Yet, Wolf is keen to stress, bioethics largely stood apart from these other developments and did little to concretely specify and assess the ethical standards it was recommending, perhaps because bioethicists were generally suspicious of ethics enforcement: “Increasingly, we see the quality of other dimensions of care routinely assessed. Ethics is not” (p. 123).

Wolf called for the core logic and methods of quality assurance to be applied to ethics. This entailed (a) specifying good quality in ethics, (b) formulating and applying measures of ethics quality, (c) using feedback loops to bring about change. The VHA’s work on what they label ‘integrated ethics’ (IE) can be seen as a full-blown realisation of this call.^11^ It represents the harnessing of QI for ethics:

IE explicitly calls on health care organizations to employ the tools of QI to systematically identify recurring ethical concerns, conduct root cause analyses, and develop systemic solutions to close “ethics quality gaps”. (p. 3)

An ‘ethics quality gap’ is the difference between prevailing ethics practices and good ethics practices. It is presented as something that can be subjected to and identified from measurement. For example, a healthcare institution may espouse norms of confidentiality or cultural sensitivity with regard to professional behaviours, or may see the transparency of its senior management board as ethically important. The IE approach involves designing measures to capture the level of adherence to such standards, and if gaps are identified, implementing interventions such as the following: ‘redesigning work processes to better support ethical practices; implementing checklists, reminders and decision support; developing specific protocols to promote ethical practices and redesigning incentive or reward systems to motivate practice in accordance with ethics standards’ (p. 5).^12^

This application of QI to ethics quality reinforces the picture of QI as a technology—as about tools and techniques; in so doing, it usefully underlines the distance between QI and the more open-ended and qualitative ways in which healthcare improvement might be discussed and in which ethics is normally discussed. It thus also raises some fundamental questions about the nature and meaning of quality (which we will turn to in the ‘Better quality healthcare or better healthcare?’ section).

It seems to us that most bioethicists will have ambivalent feelings about the account of ethics quality summarised here. On the one hand, they may readily accept the challenge that bioethics often operates too far from the specifics of practice, and therefore welcome the ambition of those who are seeking to ‘narrow the gap between ethics rhetoric and clinical action’ (p. 128).^10^ On the other hand, they are likely to be sceptical about the idea of, and measurement of, ethics quality, and about a technological approach to ethics more broadly. Such scepticism would arise from the insight that there is something inherently contentious about what counts as success in ethics and from a closely related concern about instrumentalism in ethics, that is, about a model that treats ethics *ends* as something that can be separated out from *mean*s such as incentives. For example, the VHA identifies ‘shared decision-making’ (SDM) between clinicians and patients as an important professional norm and a dimension of ethics quality. But there are deep-seated contests about what counts as SDM and about exactly how much of what kinds of SDM are ethically desirable in different contexts, for different populations and for what reasons.^13 14^ In addition, the institutional frameworks that support SDM cannot be separated from these contests. SDM practised as a result of a system of extrinsic incentives is, prima facie, not the same as SDM practised without such incentives. The same applies to other, including seemingly weaker, mechanisms such as protocols, checklists and decision support tools. We might, for instance, want to argue that, or investigate whether, protocolised SDM is less authentic and responsive than non-protocolised SDM. The chosen mechanisms are constitutive of the ends, because judgements of ethics quality are not about surface behaviours but about systems of actions and purposes that are partly constituted by broader institutional cultures.

In short, treating ethics as a suitable object for QI misapprehends the scope of ethics. Ethics—or ethical practice—cannot simply be treated as a defined end to aim towards, as the mechanisms for achieving ethical ends are also of ethical concern. Noticing the shortcomings of applying a means-ends QI model to ethics has, of course, implications for the ethics of QI more generally (discussed in the ‘Applying ethics to QI’ section). It highlights that even if we are inclined to see the centre of gravity of QI ethics as about the application of utilitarian thinking to a form of healthcare technology, we need to be very mindful of the need to balance this emphasis with attention to, and questioning of, the ethical constitution and implications of QI purposes and processes.

## Better quality healthcare or better healthcare?

Both QI and healthcare ethics are relevant to healthcare improvement, but they construct ‘better healthcare’ in very different ways.

When QI is understood as about technical interventions designed to bring about measurable improvements in healthcare, then ‘quality’ is seen as the product of, and the label for, these measurable improvements. This version of things seems to suit some facets of healthcare improvement much more than others. If we are focusing, for example, on improving the effectiveness of a particular kind of treatment then it is plausible to operationalise some criteria for effectiveness into relevant measures and equate an improvement in the measures with an improvement in that dimension of quality. The same could apply in the case of safety, operationalised as reforming specific practices to take them below a relevantly defined threshold of measurable risks and harms. However, this way of looking at things seems much less plausible in relation to ethics quality. We can certainly construct some measures of adherence to ethics standards but, we suggest, it is unlikely that we would ever view these as definitive of ethics quality: rather, at most, they might be useful but partial indicators.

In other words, technical operationalisation seems more suited to some quality-related concerns than others. But this same point could be made by contrasting different ways of understanding the nature of ‘quality’. QI largely treats quality as a measurable property; by contrast, judgements about ethics are more usually seen—in the way qualitative assessments are normally understood—as about the making of non-quantitative and perhaps non-quantifiable evaluative judgements.

The ethics quality literature thus represents a useful limiting case for QI. The contrasts it highlights apply to other areas of healthcare quality and improvement, including several that feature prominently in QI literature, such as equity and person-centredness. These are arguably among the least operationalisable dimensions of healthcare quality, yet within the QI literature—just as with ethics quality—they are also treated as the names of measurable properties.^15–17^ In other words, the same concerns apply to them as to ethics quality. And when we look more closely, the more readily operationalisable dimensions of quality, such as effectiveness, safety and timeliness, are also contested and normative,^18^ and need to be specified and contextualised to make them measurable. This suggests important limits to the operationalisation of quality more generally.

For the most part then, reference to healthcare ‘quality’ within QI picks out some (combination of) measurements. Claims that something will result in ‘better quality healthcare’ are situated within technicist discourses—with quality as a measurable property that is increased by systematic intervention (understood on a causal model close to that applied in biomedical reasoning). Indeed, QI can be seen as parallel to, or an extension of, a specific (narrow) conception of biomedical model thinking: as biomedical interventions bring about health outcomes, so QI is supposed to bring about more and better outcomes.

By contrast, claims that something will result in ‘better healthcare’ need not be technicist—they can instead reflect an overall evaluative judgement that something is better than a comparator. Given this account, we can say that the word ‘quality’ both adds and subtracts from the idea of ‘better healthcare’—it adds a level of specification but at the same time disguises the inherent contestability of quality dimensions (including questions about which dimensions matter and about how these should be understood). In particular, the technicist quality discourse replaces (a) the presumption that assessments of ‘better healthcare’ are compatible with, and will almost always include, high levels of uncertainty and disagreement about healthcare purposes and possibilities, with (b) the presumption that it is possible, at least in principle, to close down the scope of contestation through more precise definitions and measurements.

It is important to stress that there can be broader constructions of healthcare improvement than the technological model of QI that we have emphasised. These alternatives encompass ‘enlightenment’ as well as ‘engineering’ models of change—for example, recognising the importance of diffuse cultural adaptation and of various kinds of learning for healthcare actors.^19^ Nonetheless, these other constructions are usually anchored in approaches that operationalise quality because of the widespread and understandable commitment to the idea that responsible improvement activities must be based around carefully specified—and hence measurable—definitions of the relevant dimensions of quality whose improvement is being sought.

QI ethics has thus to begin with the recognition of the hybrid nature of QI. It is a field that is elastic (and wise) enough to include relatively open-ended and obviously value-laden ideas such as equity and person-centredness within its remit, but which is, at the same time, basically committed to seeing quality as a measurable property.

## Towards an ethics of healthcare improvement

A sufficiently ambitious ethics of healthcare improvement must tackle a large range of concerns. Cutting across these concerns, we have been suggesting, are critical questions about how we think about healthcare improvement including how to frame the ethics of improvement. In particular, we need to be mindful of the way QI approaches to improvement are inflected by the predominance of the ‘measurable property’ conception of quality. This makes the ethics of measurement and operationalisation central to QI ethics.

This is not an abstract matter. Colleagues working at any level of a healthcare system, with an interest in better healthcare, have to decide how to pursue that interest responsibly. There are a lot of advantages to operationalising quality. First, there are crucial pragmatic questions: when you are working in a context dominated by the biomedical model, especially in an era of evidence-based policy, you are likely to be seen as both more persuasive and accountable if you are in a position to define and measure quality and QI. Second, these pragmatic advantages may coincide with more intrinsic advantages that arise from clarity, transparency and empirical testability. The actions of someone operating within technicist discourses of quality are therefore likely to be seen by many as more intelligible and legitimate and may therefore be more effective in motivating and sustaining change.

On the other hand, there are aspects of healthcare improvement where operationalisation of quality and QI is more problematic in principle and can potentially create problems in practice. For example, the notion that healthcare can be made more patient-centred by developing, applying and monitoring measures of patient experience is highly problematic. There are multiple competing candidates for what counts as good patient experience, and there is little evidence that applying such measures works to improve care on their own terms.^20^ Moreover, in some cases reliance on measures that lack credibility as indicators of patient-centredness may arguably undermine that aspect of quality as much as promote it. In very many cases the claim that some instance (some practice, some service) of healthcare has become better will be highly complex and inherently contestable. In these cases, pointing to a change in some set of measures may be relevant but is unlikely to be decisive. Rather we would be inclined to turn to a combination of quantitative and qualitative evidence and normative argumentation to determine whether x or y is better and, moreover, not to treat any single piece of evidence as conclusive in revealing whether healthcare has been improved.^21^ Of course some operationalisation will be possible in these cases but it is likely to distort or obscure important value questions. In other words, the surface ‘transparency’ of operationalisation can come at a cost, including a loss of transparency about why and how specific interpretations of quality have been selected from a repertoire of possible interpretations.

It is equally possible to shift emphasis in the other direction—that is, to apply the notion that quality judgements are contestable evaluative judgements to core areas of quality such as effectiveness. For example, if we were to adopt the plausible notion that what counts as ‘effective’ is sometimes a function of what matters to individual people, this would take us in that direction. It may, at least sometimes, be a way of introducing greater responsiveness and sensitivity into assessments of effectiveness but it also undoubtedly creates challenges for measurement and comparability and thus overall for practicability.

Thus one of the core, but neglected, ethical questions the field of healthcare improvement faces is how we should strike a balance between the two approaches to thinking about quality-related assessments—‘measurable property’ or ‘evaluative judgement’ approaches—that we have highlighted. As well as providing an important agenda for healthcare ethics this also suggests the need to enlarge the field of ‘improvement science’ so that it more explicitly incorporates and addresses normative issues.^22^ Building greater capability in this interdisciplinary area is an academically interesting project and is of great practical importance if claims about ‘improvements’ are to be rigorously grounded.

More broadly, and in conclusion, we are arguing that healthcare improvement—even understood in the relatively narrow sense as the quasi-professional domain of QI—is a very complex and diverse field, which merits an equally rich field of ethical analysis. As we have noted, there are multiple conceptions and dimensions of ‘quality’, and hence contests about how to interpret, prioritise and combine different dimensions, such as effectiveness or person-centredness. In addition, there is a wide variety of (sometimes competing, sometimes complementary) QI methodologies which have partly arisen out of the adoption and adaptation of industrial models (eg, Total Quality Management, Lean, Six Sigma, etc^23^) and partly from a range of behavioural and social science developments.^24^ This means there are a multitude of questions about the ethical defensibility and merits of diverse improvement methods, and the specific challenges and dilemmas they pose for different kinds of actors—system leaders, managers, health professionals, patients and communities and so on—working at different levels of healthcare systems. Finally, the ethics of QI needs to be seen in the context of broader conceptions of, and debates about, healthcare improvement and needs to attend to the inherent ethical constitution of all improvement activities,^25^ and the ways in which improvement purposes and practices are necessarily saturated with ethical contestation.
